# Short-Circuit Current in Polymeric Membrane-Based Thermocells: An Experimental Study

**DOI:** 10.3390/membranes11070480

**Published:** 2021-06-28

**Authors:** V. María Barragán

**Affiliations:** Department of Structure of Matter, Thermal Physics and Electronics, Faculty of Physics, Complutense University of Madrid, 28040 Madrid, Spain; vmabarra@ucm.es

**Keywords:** ion-exchange membrane, thermoelectricity, energy conversion, short-circuit current, thermocell

## Abstract

Thermocells are non-isothermal electrochemical cells used to convert thermal energy into electricity. In a thermocell, together with the ion flux, heat is also transferred, which can reduce the temperature gradient and thus the delivered electric current. A charged membrane used as a separating barrier in the electrolyte liquid could reduce this problem. Therefore, the use of ion-exchange membranes has been suggested as an alternative in terms of thermoelectricity because of their high Seebeck coefficient. Ion transfer occurs not only at the liquid solution but also at the solid membrane when a temperature gradient is imposed. Thus, the electric current delivered by the thermocell will also be highly dependent on the membrane system properties. In this work, a polymeric membrane-based thermocell with 1:1 alkali chloride electrolytes and reversible Ag|AgCl electrodes at different temperatures is studied. This work focuses on the experimental relation between the short-circuit current density and the temperature difference. Short-circuit current is the maximum electric current supplied by a thermocell and is directly related to the maximum output electrical power. It can therefore provide valuable information on the thermocell efficiency. The effect of the membrane, electrolyte nature and hydrodynamic conditions is analysed from an experimental point of view.

## 1. Introduction

When a conductive material is subjected to the simultaneous presence of thermal and electrical potential gradients, a number of thermoelectric effects may occur [1,2]. Thermoelectric effects arise from the coupling of two thermodynamic potentials, the electrochemical potential and the temperature. In a thermoelectric system, this coupling allows the conversion of thermal energy into electricity. This conversion has been well known for a long time. When a conductive material is subjected to a thermal gradient, charge carriers migrate along the gradient from hot to cold. In the open-circuit condition, charge carriers will accumulate in the cold region, resulting in the formation of an electric potential difference, known as Seebeck potential.

Thermoelectric materials have been subjected to research [1,3]. Conventional thermoelectric materials utilize electron diffusion under temperature gradients [1]. Seebeck effect in metals and semiconductors has been well explored. On the other hand, thermo-galvanic cells operate with liquid electrolytes [4,5]. Recently, other materials have been considered as thermoelectric materials [6]. Ion-exchange membranes have been suggested as alternative to semiconductors. Seebeck coefficients of the order of mVK^−1^ have been reported with ion-exchange membranes depending on the experimental conditions [7]. They have lower manufacturing cost and may have the capacity to produce electricity from low grade waste heat [8,9]. It was found that heterogeneous ion-exchange membranes have larger thermoelectric potentials in general than homogeneous ion-exchange membranes, both cationic and anionic [10,11]. Moreover, in non-isothermal electrochemical cells, also named thermocells, electrode choices and electrolyte conditions can help increase the value of the Seebeck coefficient [7].

The interest in membrane-based systems to convert low grade heat into electricity is increasing [9,12,13,14]. Jokinen et al. [15] carried out the first experimental studies into thermoelectric power generation using ion-exchange membranes, using a cell with Ag|AgCl electrodes at the same temperature. The thermal efficiency of a thermocell with hydrogen gas electrodes and a proton exchange membrane was modelled by T. Marquardt et al. [16]. Kim et al. [9] studied the thermoelectric effect comparing behaviours of three different types of solid-state polyelectrolytes. Despite growing interest, studies of the output electrical power in membrane-based thermocells are still scarce and most of them refer to open-circuit configuration to estimate the Seebeck coefficient [7].

Although the values for the output electrical power found in the literature are still low, an optimization of the system could improve the efficiency of the thermoelectric conversion of membrane-based thermocells. Moreover, the thermoelectric properties of ion-exchange membranes may be used to increase the efficiency of other membrane processes for energy conversion such as reverse electrodialysis [14]. In recent years, the interest in micro- and nano-thermoelectric generators has increased and many studies are moving towards the use of nanostructured materials. In terms of power constraint, studies have suggested that power sources in the range of 10^−6^ Wcm^−2^ would be ideal for biomedical applications [17,18].

One important issue in thermocells is the dependence of the maximum output power on the temperature difference. This depends both on the open-circuit voltage and the short-circuit current [1]. A linear dependence between the open-circuit voltage and the temperature difference is commonplace in reported thermocells. However, precedent results in the literature demonstrate significant non-linearity between short-circuit current density and temperature difference in some thermo-galvanic cells [19].

This work aims to contribute to the study of membrane-based thermocells. For this purpose, the relation between short-circuit current and temperature difference has been investigated from an experimental point of view in a basic ion-exchange membrane-based thermocell.

## 2. The Membrane-Based Thermocell

Consider the following non-isothermal electrochemical cell:

(1) $$\underset{T_{1}}{\underbrace{\mathrm{Ag}\left(s\right)\left|\mathrm{AgCl}\left(s\right)\right|\mathrm{XCl}\left(\mathrm{aq}.\;c_{0}\right)}}\left|\mathrm{ion}-\mathrm{exchange}\;\mathrm{membrane}\right|\underset{T_{2}}{\underbrace{\mathrm{XCl}\left(\mathrm{aq}.\;c_{0}\right)\left|\mathrm{AgCl}\left(s\right)\right|\mathrm{Ag}\left(s\right)}}$$

Two Ag|AgCl electrodes connect two half cells with equal concentration *c*_0_ of a XCl electrolyte solution, but at different temperatures. Both aqueous solutions are separated by an ion-exchange membrane. In a non-isothermal electrochemical system, associated with heat transport, there is also charge and mass transport [20]. The temperature gradients drive electrolyte and thermo-osmotic water transport through the membrane. The complete cell has distinct phases, two connecting leads from electrodes to an external circuit, anode- and cathode-surfaces, two bulk electrolyte solutions, a membrane, and two interfaces between the membrane and the solutions. In an open-circuit configuration, the measured cell potential will be the sum of the thermal potential of the membrane system and the contribution of the electrodes [21]. For highly charged membranes, the major contribution to the thermal membrane potential arises from the temperature dependence of the Donnan potentials, as the electrolyte diffusion through the membrane is practically negligible due to the low membrane permeability for co-ions.

**Figure 1:**
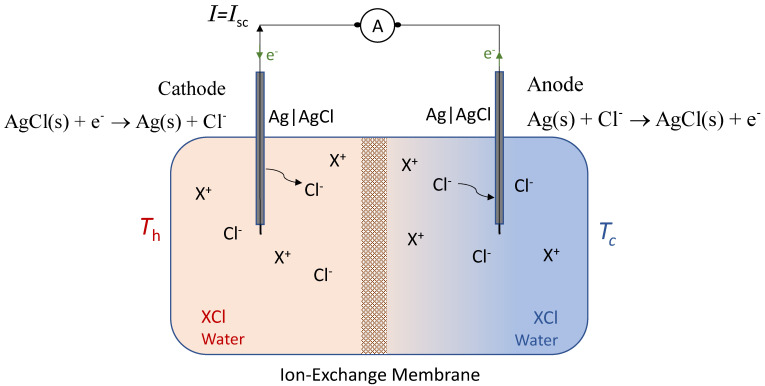
Schematic representation of a XCl-H_2_O thermocell with an ion-exchange membrane and Ag|AgCl electrodes in the short-circuit configuration. A: Amperemeter; *I*_sc_: Short-circuit current; *T*_h_: Hot temperature; *T*_c_: Cold temperature.

In a short-circuit configuration (Figure 1), with a closed circuit, ions are current carriers in the electrolyte through the membrane, chemical reactions will take place at the electrodes, and electrons take over as current carriers through the electronic conductors. During the passage of current through the cell, matter is transferred from one electrode to the other as a result of the electrochemical reactions at the electrode/electrolyte interfaces and ion transport in the electrolyte and in the membrane. The electrodes are short-circuited, and the electric potential difference equals zero; in the ideal case there is no resistance to an electric current passing through the outer circuit.

The chemical reactions at the electrodes are as follows:

(2) $$\begin{array}{l} {\mathrm{Cathode}:\;\mathrm{AgCl}(s)+e}^{-}\to {\;\mathrm{Ag}(s)+\mathrm{Cl}}^{-}\\ {\mathrm{Anode}:\;\mathrm{Ag}(s)+\mathrm{Cl}}^{-}\to {\;\mathrm{AgCl}(s)+e}^{-} \end{array}$$

If the effect of thermo-osmosis on the ion flux density is neglected, the current inside the membrane will be carried by the flux of ionic species inside the membrane [22]. For ideal ion-exchange membranes, with a complete exclusion of co-ions, this flux will only be due to counter-ions, and the current inside the membrane will be exclusively carried by counter-ion migration.

**Figure 2:**
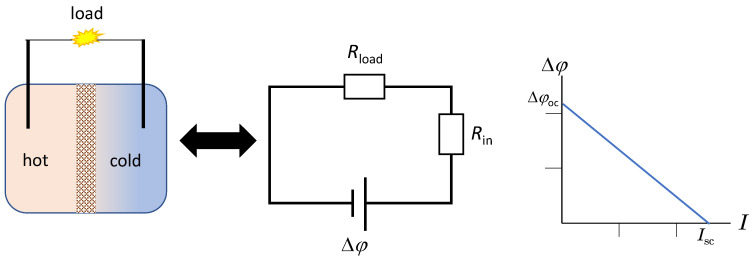
Sketch of a basic membrane-based thermocell and equivalent circuit (on the left), and characteristic Δ*φ*-*I* curve (on the right). Δ*φ*: Output voltage; *R*_in_: Internal resistance; *R*_load_: Load resistance; Δ*φ*_oc_: Open-circuit voltage; *I*_sc_: Short-circuit current.

If we consider the non-isothermal electrochemical cell as a thermo-generator, with a material inserted between a hot and a cold temperature, from the electrical point of view the system is equivalent to a voltage generator (Figure 2) with a supply voltage, Δ*φ*, and an internal resistance, *R*_in_, and the intensity of the electric current, *I*, through the overall circuit will be [1]:

(3) $$I=\frac{\Delta \varphi }{R_{\mathrm{in}}+R_{l\mathrm{oad}}}$$

where *R_l_*_oad_ is the load resistance.

The internal resistance will be the sum of the membrane system (membrane and electrolyte), electrodes and external electric circuit resistances.

For typical membrane-based thermocells, it has been experimentally demonstrated that the open-circuit voltage is proportional to the temperature difference Δ*T* [7,10,11,15,19]. In addition, the experimental characteristic curves for typical thermocells are linear, indicating that the internal resistance is independent of temperature difference. In this case, $\Delta \varphi =\Delta \varphi_{\mathrm{oc}}-IR_{\mathrm{in}}$, where Δ*φ*_oc_ is the open-circuit voltage.

In short-circuit configuration, the short-circuit current can be expressed by [1]:

(4) $$I_{\mathrm{sc}}=\frac{\Delta \varphi_{\mathrm{oc}}}{R_{\mathrm{in}}}$$

The short-circuit current indicates the upper bound of the electric current delivered by the thermocell.

The output electrical power, *P* = Δφ*I*, obtains its maximum value when the load resistance equals internal resistance. This maximum value can be expressed as:

(5) $$P_{\max}=\frac{\Delta \varphi_{\mathrm{oc}}^{2}}{4R_{\mathrm{in}}}=\frac{R_{\mathrm{in}}I_{\mathrm{sc}}^{2}}{4}$$

From Equation (4), a linear relation *I*_sc_ ∝ Δ*T* should also be expected between the short-circuit current delivered by the thermocell and the established temperature difference.

The maximum output electrical power density can be expressed as:

(6) $$p_{\max}=0.25\Delta \varphi_{\mathrm{oc}}j_{\mathrm{sc}}$$

Consequently, a maximum output electrical power density quadratic in the temperature difference *p*_max_ ∝ Δ*T*^2^ would be expected if linear dependences in the temperature difference are considered both for open-circuit voltage and for short-circuit current density.

## 3. Experimental

### 3.1. Materials

Different commercial ion-exchange membranes were used in this work. Three cation-exchange membranes: CR61-CZL-412 (hereafter named CR61), CR65-AZL-412 (hereafter named CR65) and CR67-HMR-412 (hereafter named CR67), and one anion-exchange membrane: 103-QZL-386 (hereafter named 103QZL). All are highly selective heterogeneous membranes manufactured by Ionics, Incorporated (Watertown, MA, USA). Table 1 presents some relevant properties of the membranes.

Ionics’s heterogeneous membranes are ionic selective membranes comprising cross-linked sulfonated copolymers of vinyl compounds. The membranes are homogeneous films cast in sheet form on synthetic reinforcing fabrics. Modacrylic polymer (copolymer of vinyl chloride and acrylonitrile) is the fabric used for cationic CR61 and CR65 and anionic 103QZL membranes, and acrylic for the cationic CR67 membrane. The ionic fixed sites are sulfonic acid groups for the cation-exchange membranes, and quaternary ammonium groups for the anion-exchange membrane.

Aqueous solutions of lithium, sodium, potassium, and cesium chloride were used as electrolytes. Pure pro-analysis grade chemicals and distilled pure water were used. Before measurements were carried out, the solutions were degassed to prevent bubble formation during the measurement process.

### 3.2. Method

**Figure 3:**
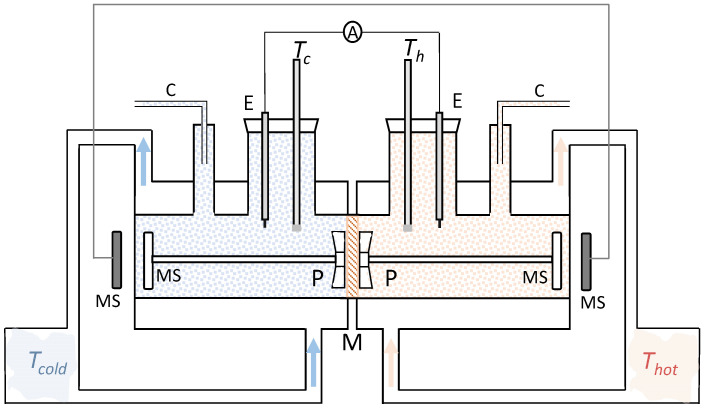
Sketch of the experimental device used in this work. M: Ion-exchange membrane; P: Propeller, MS: Magnetic stirrer; C: Capillary; E: Ag|AgCl electrode; *T_i_*: Platinum resistance thermometer; A: Amperemeter.

The device used in this work was similar to those used in previous works to measure thermal membrane potential [23,24]. A general sketch of the experimental device is shown in Figure 3.

The cell consisted essentially of two equal cylindrical glass chambers, each having a large enough volume to ensure that the concentration changes in the bulk solutions during the measurements were negligible. Each chamber possessed a jacket, also made of glass, where water, maintained at constant temperature, circulated by means of a TV-16 A thermostat. It was ensured that the fluctuations in temperature in the thermostatic liquid were lower than 0.1 K. The solutions in both chambers could be stirred by a chain-driven cell magnetic stirrer assembly which enabled the selection of a stirring rate of between 0 and 375 rpm.

The membrane was held vertically between the two chambers. The membrane surface area exposed to the flow was 0.793 cm^2^. One shaped capillary tube was introduced in each chamber, in such a way that the horizontal portions of the tubes were at the same height, to prevent pressure differences between the two chambers. The temperature of each chamber was measured with platinum resistance thermometers placed inside the chamber for this purpose. The resistance was measured with a digital Keithley 199 System DMM/SCANNER multi-meter and recorded during the experiment. The thermometers were previously calibrated to obtain the resistance-temperature curves. One Ag(s)|AgCl(s) electrode was placed inside each chamber to measure the electric current. This consisted of a linear Ag wire of approximately 10-mm longitude and 1-mm diameter, prepared by the usual method [25]. The electric current was measured as a function of time by means of a Keithley 195 System DMM with a resolution of 100 pA in the 20 µA range and recorded for a 4–5 h period. The electrodes were connected to the amperemeter in such a way that the electrode corresponding to the solution at the lower temperature was grounded.

Before the membrane was positioned in the cell, it was equilibrated with the electrolyte solution to be used, by immersing it in the solution for a minimum of 36 h. Once the selected temperatures were stabilized in the corresponding baths, they were circulated through the corresponding jackets and a temperature difference was established between both sides of the membrane.

This process was reiterated by progressively increasing the established temperature difference and always maintaining the same mean temperature.

## 4. Results and Discussion

### 4.1. Curves (I_sc_-t)

**Figure 4:**
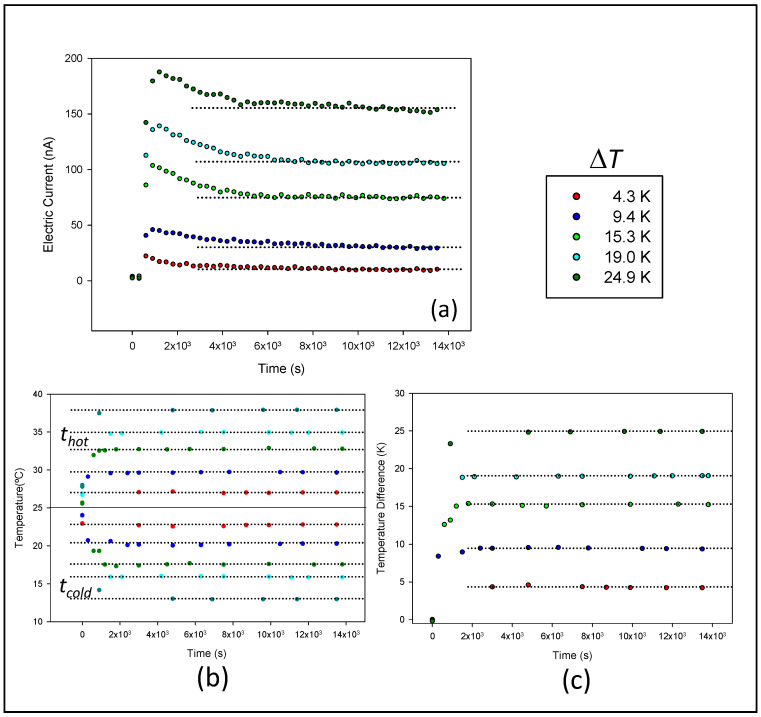
Short-circuit current (**a**), hot and cold temperatures (**b**), and temperature difference Δ*T* (**c**) as a function of time. The data correspond to membrane CR61, with NaCl 10^−4^ M, natural convection and a mean temperature of 25 °C.

Short-circuit current-time curves were measured for different membrane systems. Similar results were obtained for all the studied cases. When the temperature difference was established, an electric current appeared in the system. Figure 4 presents, as an example, the results obtained for membrane CR61 with 10^−4^ M NaCl as electrolyte under natural convection condition.

After an initial transitory trend, a stable value of the current was measured. When power is generated in the thermocell, concentration gradients rapidly form within the cell and current drops, before eventually continuous steady-state outputs are achieved. This can take different times [19,26]. In the investigated thermocell, this time depended on the membrane system, but in general it took around 1 h for the system to reach a stable current value. This stable value was maintained throughout the measurement time. Corresponding short-circuit current value was then estimated as the average of the values obtained in the considered stable interval, and the standard deviation was indicated as error in the measure. The relative measurement uncertainty in each experiment varied by less than 10% for the lowest current and less than 1% for the highest current. As it was expected, in all the investigated cases, the short-circuit current increased when the temperature difference was increased.

An inversion of the temperature difference between both chambers was found to result in a current inversion. This is important for maintaining the state of the electrodes.

### 4.2. Short-Circuit Current Density vs. Temperature Difference

#### 4.2.1. Effect of the Membrane

**Figure 5:**
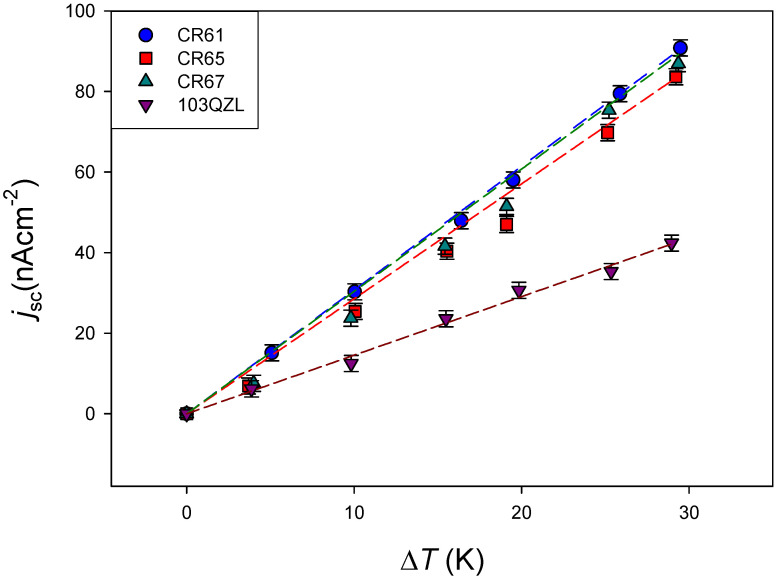
Short-circuit current density versus temperature difference for the different ion-exchange membranes. Electrolyte: 10^−4^ M KCl. Mean temperature: 25 °C. Natural convection. Lines correspond to linear fits of the experimental data.

Figure 5 presents the values obtained for the short-circuit current density as a function of the temperature difference with the different tested membranes. Under the considered experimental conditions, a similar behaviour was observed with all membranes, and a linear relation between short-circuit current density and temperature difference was observed.

From the slope of the corresponding linear fit, the short-circuit current density per K of temperature difference has been obtained for each investigated membrane. The results are shown in Table 2.

These results show that there was no significant difference among the tested cationic membranes. This is an expected result given the similar electrical properties of the membranes. A lower value was obtained for the anion-exchange membrane, perhaps due to the different transport entropy for ion Cl^−^ [27]. Typical values for the Seebeck coefficient for ion-exchange membranes are in the order of magnitude of 1 mVK^−1^ [7]. The low observed electric current value was probably due to the high value of the thermocell internal resistance. Considering the data shown in Table 1, low membrane resistances are expected for the used membranes, and the greatest contribution to the cell internal resistance was probably due to the electrolyte contribution, which could be reduced, decreasing the distance between the electrodes [28] or increasing the electrolyte concentration [15].

#### 4.2.2. Effect of the Electrolyte

**Figure 6:**
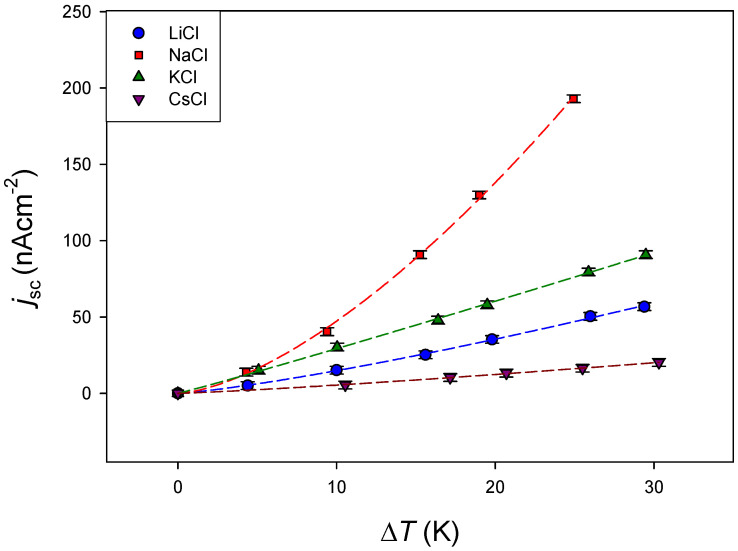
Short-circuit current density versus temperature difference for CR61 at different 10^−4^ M electrolytes and natural convection. Mean temperature: 25 °C. Lines corresponds to the fit of the experimental data to Equation (7).

Membrane CR61, with higher short-circuit current values, was selected to investigate the effect of the electrolyte nature. LiCl, NaCl, KCl and CsCl aqueous solutions with 10^−4^ M concentration were used. The values obtained for short-circuit current density as a function of the temperature difference are shown in Figure 6.

With the exception of NaCl, a linear relation was observed between the short-circuit current density and the established temperature difference. From the slops of the linear fits of the experimental data in the linear interval, the values of the short-circuit current density per K were obtained for each electrolyte. These values are shown in Table 3.

In general, a negative correlation with the cation size was observed, like that observed in Seebeck coefficient in similar membrane systems [23,29], and the short-circuit current density decreased with increase in the cation size. However, LiCl deviated from this correlation, presenting a lower value than NaCl and KCl, but different trends have been also found in the literature depending on the membrane type [7]. Moreover, a lower transport entropy was estimated for Li^+^ than for the other cations in aqueous electrolyte solutions at 25 °C and 0.01 M using thermocells with Ag/AgCl electrodes [27]. It should be pointed out that a non-linear dependence was observed between short-circuit current density and the temperature difference in the case of NaCl. Precedent results in the literature also demonstrated significant non-linearity in *j_sc_*-Δ*T* curves in thermo-galvanic cells. Buckingham and Aldous [19] showed an excellent correlation between the short-circuit current density and the temperature difference by fitting a power trendline between the two variables according to the equation:

(7) $$j_{\mathrm{sc}}=\alpha \Delta T^{\beta }$$

where *α* and *β* were fitting parameters. They employed a thermocelll with a K_3/4_[Fe(CN)_6_] solution contained by a PMMA cell with Pt electrodes, obtaining the best fit for *β* = 1.3.

A power law model has been checked for the membrane-based thermocell investigated in this work, fitting *j*_sc_ vs. Δ*T* to Equation (7). An excellent correlation was also found. The values of parameters *α* and *β* obtained for the different electrolytes are shown in Table 4.

LiCl and NaCl largely deviated from the linear dependence. The reason of this trend is not clear. It could be related to the electrode kinetic. Buckingham et al. [30] found that the ratio of reduced:oxidized species in thermocells can be excellently modelled by the Butler-Volmer equation. Salazar et al. [28] also noticed the importance of the kinetic at the electrodes to thermocell efficiency, but an exponential relationship should have been observed in this case. Further work is necessary to understand the origin of this power trend.

#### 4.2.3. Effect of the Solution Stirring Rate

**Figure 7:**
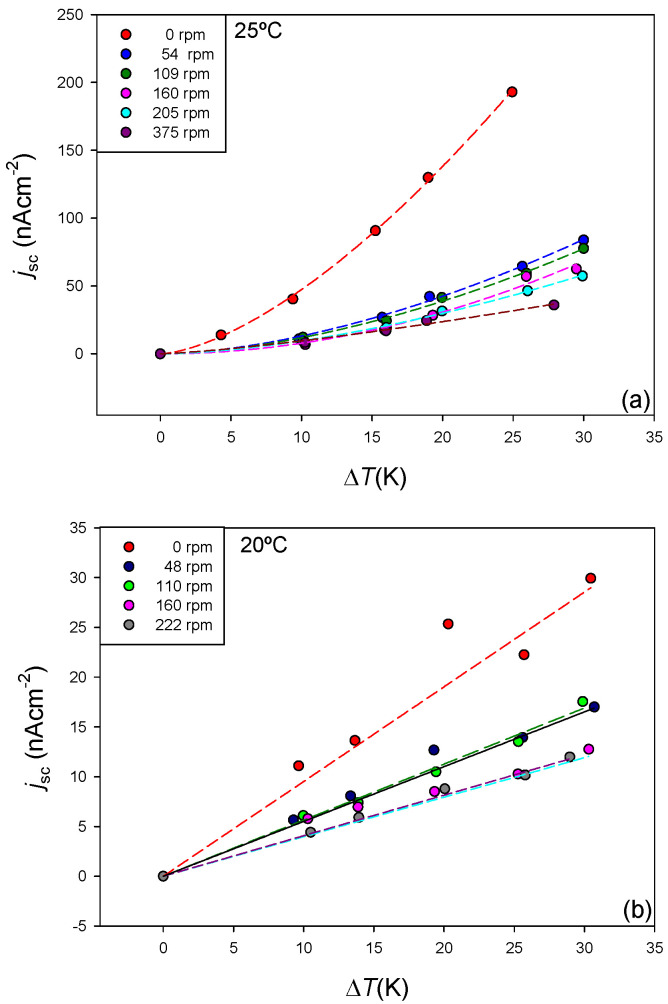
Short-circuit current density versus solution stirring rate for CR61 under different conditions: (**a**) Mean temperature: 25 °C; Lines corresponds to fit the experimental data to Equation (7). (**b**) Mean temperature: 20 °C. Lines correspond to linear fits.

The significant effect that polarization effects have in the membrane processes is known [31]. Temperature polarization affects the temperature difference between both sides of the membrane when the Seebeck coefficient is estimated [23,24]. Under natural convection conditions, it is also expected that concentration polarization occurs, originating a concentration gradient that can contribute to the movement of the ionic species through the membrane. For the purpose of analysing the effect of the polarization effects in the short-circuit current, different experiments were carried out, stirring the solutions in contact to the membrane surfaces. The use of forced convection to reduce polarization effects is common in membrane systems [11,23,32]. Two experimental situations were analysed for the 10^−4^ M NaCl-CR61 membrane system, changing the mean temperature of the system. The results are presented in Figure 7. It can be observed that the effect of forced convection was a decrease of the short-circuit current density value with respect to those obtained under natural convection conditions.

The stirring process reduces the thickness of the temperature polarization layers at both side of the membrane [7,11]. The effective temperature difference would be lower under natural convection conditions and thus an increase of the short-circuit current value would be expected with increased stirring. However, the concentration gradient established at both sides of the membrane as a consequence of the concentration polarization effect may also be reduced by the stirring process. The existence of a concentration difference at both sides of the membrane may contribute to both ion diffusion and water transport convection. In this case, it would indicate that the observed electric current would not only be due to the thermal gradient. The formation of concentration polarization layers could also affect the internal resistance of the system. Further work is necessary to clarify the observed results.

The effect of the stirring rate in the linear relationship between short-circuit current density and temperature difference has been investigated. To this purpose, Equation (7) was rewritten as:

(8) $$\frac{j_{\mathrm{sc}}}{\Delta T}=\alpha \Delta T^{\beta -1}=\alpha \Delta T^{\beta \prime }$$

Parameter *β*′ = *β* − 1 will be close to zero for linear trend between short-circuit current density and temperature difference. Table 5 presents the results obtained with 10^−4^ M NaCl and a mean temperature of 25 °C.

At a mean temperature of 20 °C, the experimental results were worse when modelled using Equation (8), and parameter *β*′ was always lower than 10^−10^, which indicated that a linear relation could be supposed at this mean temperature. Table 6 presents the linear fit parameters for data corresponding to 10^−4^ M NaCl and a mean temperature of 20 °C.

Under the two investigated experimental conditions, a forced convection seems to decrease the value of the short-circuit current density, but no significant trend was observed with the stirring rate value.

It can be noticed that an increase of the mean temperature in the thermocell increases the short-circuit current density and contributes to the loss of linearity in the curves *j*_sc_-Δ*T*. In general, the non-linear trend seems to be favoured by large values of the short-circuit current. Further work is necessary to clarify this behaviour.

### 4.3. Molar Flux Density of Ionic Species

Electric current at the electrodes must be maintained by an ion flux in the thermocell. For a highly selective membrane, the counter-ion transport number is practically the unity, and the electric current through an ion-exchange membrane will be transported basically by counter-ions. Thus, the molar flux density of ionic species moving through the membrane in the thermocell can be estimated from the corresponding short-circuit current value, according to the expression *J*_i_ = *I*_sc_/z_i_F, where *z*_i_ is the ion valence and F is the Faraday constant. In the absence of concentration gradients or convection, this flux would be due to thermal diffusion and it would give information about the Soret coefficient of the counter-ion in the membrane phase. The values obtained with KCl 10^−4^ M under natural convection were of the order of 10^−10^ mols^−1^m^−2^K^−1^ for all the tested membranes, although these values should be considered as effective values taking into account possible diffusion and convection contributions. From the data presented in Table 6, it can be noticed that a stirring of the solutions in contact with the membrane surfaces can reduce the molar flux through the membrane, and then the Soret effect, by more than half. Although the diffusive boundary layer at the electrodes has been shown to limit the current densities of thermocells [28], forced convection lowers the electric current delivered by the tested membrane-based thermocell.

## 5. Conclusions

Membrane-based thermocells in short-circuit configuration were investigated. Short-circuit current density values of the order of 10^−5^–10^−6^Am^−2^ per K were obtained using heterogeneous highly charged polymeric membranes and alkali chloride electrolyte solutions, reversible Ag|AgCl electrodes at different temperatures, and natural convection conditions. A stable electric current value was reached after approximately one hour after stablishing the temperature gradient and maintaining this for more than 5 h.

No significant differences were observed among different cation-exchange membranes with similar electrical properties in KCl 10^−4^ M solutions. A lower value of the short-circuit current density was obtained with the anion-exchange membrane under the same experimental conditions, probably due to the different transport entropy for counter-ion Cl^−^.

The effect of the cation of the alkali chloride for the more selective cation-exchange membrane and electrolyte concentration of 10^−4^ M was investigated. The highest short-circuit currents were obtained with NaCl. The value decreased with the cation size, although LiCl deviated from this correlation, presenting a lower value than NaCl and KCl. Lower short-circuit current density values were obtained at the lower mean temperature.

The influence of solution stirring was analysed. The effect of forced convection was a decrease in the short-circuit current density value with respect to those obtained under natural convection conditions. This could be due to a favourable contribution of the concentration gradient caused by polarization effects to the observed electric current, but further work is necessary to clarify the observed trend. The dependence of short-circuit current density on the temperature difference was analysed. A non-linear trend was observed in some of the tested systems. In general, the non-linear trend seems to be favoured by larger values of the short-circuit currents. An excellent correlation between the short-circuit current density and the temperature difference by fitting a power trendline was observed in the non-linear systems.

Although low values of the short-circuit current were obtained, a decrease of the distance between the electrodes in the thermocell or higher electrolyte solutions could reduce the internal resistance A value of half the corresponding short-circuit current density value would be expected for the current delivered by the thermocell at its maximum power. It would be interesting to use as micro-generator in small power density applications.

## Figures and Tables

**Table 1:** Properties of selected commercial membranes.

Membrane	Ion-Exchange Capacity (meq/dry Gram Resin)	Area Specific Resistance * (Ω cm^2^)	Dry Thickness (10^−3^ m)	Water Content (%)
CR61	2.7	13	0.60	43
CR65	2.3	8.1	0.61	56
CR67	2.1	10.1	0.57	70
103QZL	2.1	15	0.63	46

* In 0.01 N NaCl.

**Table 2:** Short circuit density per K of temperature difference for the different ion-exchange membranes. Values correspond to linear fits of the experimental data in Figure 5.

Membrane	$j_{\mathrm{sc}}/\Delta T\;(10^{-6}{\mathrm{Am}}^{-2}K^{-1})$	*r*
CR61	30.7 ± 0.3	0.999
CR65	28.5 ± 1.0	0.991
CR67	30.4 ± 0.11	0.990
103QZL	14.5 ± 0.5	0.996

**Table 3:** Short circuit density per K of temperature difference for the different electrolytes. Values correspond to linear fits of the experimental data shown in Figure 6.

Electrolyte	$j_{\mathrm{sc}}/\Delta T\;(10^{-6}{\mathrm{Am}}^{-2}K^{-1})$	*r*
LiCl	19.9 ± 0.9	0.984
NaCl	43 ± 4	0.987
KCl	30.7 ± 0.03	0.999
CsCl	6.7 ± 0.2	0.990

**Table 4:** Parameters *α* (10^−5^Am^−2^K^−β^) and *β*, and regression coefficient *r* (from fitting the experimental data in Figure 6 to Equation (7)) for the different electrolytes.

Electrolyte	*α*	*β*	*r*
LiCl	0.83 ± 0.08	1.26 ± 0.03	0.9995
NaCl	1.35 ± 0.13	1.54 ± 0.03	0.9997
KCl	2.65 ± 0.15	1.04 ± 0.02	0.9995
CsCl	0.35 ± 0.04	1.19 ± 0.03	0.9995

**Table 5:** Parameters *α* (10^−5^Am^−2^K^−1^) and *β’*, and regression coefficient *r* (from fitting experimental data in Figure 7a to Equation (8)) at 25 °C.

rpm	*α*	*β′*	*r*
0	1.38 ± 0.13	0.54 ± 0.04	0.998
54	0.21 ± 0.05	0.76 ± 0.07	0.995
109	0.22 ± 0.05	0.72 ± 0.07	0.995
160	0.06 ± 0.04	1.09 ± 0.15	0.99
205	0.19 ± 0.03	0.70 ± 0.06	0.997
375	0.32 ± 0.13	0.43 ± 0.13	0.99

**Table 6:** Short-circuit current density per K of temperature difference at a mean temperature of 20 ^o^C. Values correspond to linear fits of the experimental data in Figure 7b.

rpm	$j_{\mathrm{sc}}/\Delta T\;(10^{-6}{\mathrm{Am}}^{-2}K^{-1})$	*r*
0	0.95 ± 0.07	0.96
48	0.55 ± 0.03	0.99
110	0.56 ± 0.04	0.996
160	0.40 ± 0.03	0.97
222	0.41 ± 0.01	0.998
